# Osteogenic differentiation by pre-osteoblasts is enhanced more on 3D-PRINTED poly-ɛ-caprolactone scaffolds coated with collagen and hydroxyapatite than on poly-ɛ-caprolactone/hydroxyapatite composite scaffolds coated with collagen

**DOI:** 10.1177/08853282251392820

**Published:** 2025-10-28

**Authors:** Ali Moghaddaszadeh, Mohammad Ehsan Ghiasvand, Hadi Seddiqi, Sonia Abbasi-Ravasjani, Jenneke Klein-Nulend

**Affiliations:** 1 Department of Biomedical Engineering, Science and Research Branch, 125643 Islamic Azad University, Tehran, Iran; 2 Department of Mechanical Engineering, 48410 Amir Kabir University of Technology, Tehran, Iran; 3 Department of Oral Cell Biology, 1192 Academic Centre for Dentistry Amsterdam (ACTA), University of Amsterdam and Vrije Universiteit Amsterdam, Amsterdam Movement Sciences, Amsterdam, The Netherlands

**Keywords:** bone tissue engineering, collagen, finite element modeling, hydroxyapatite, poly-є-caprolactone, osteogenic differentiation, 3D-printed scaffolds

## Abstract

Three-dimensional (3D)-printed poly-ε-caprolactone (PCL) scaffolds lack sufficient bioactivity for optimal bone tissue engineering applications. This shortcoming can be overcome by coating PCL scaffolds with collagen and hydroxyapatite (PCL/col-HA) or by applying a collagen coating to PCL-HA composite scaffolds (PCL-HA/col). Here we aimed to test which type of scaffold is more effective in stimulating osteogenic activity. Moreover, the scaffolds’ physicomechanical properties were characterized. 3D-printed PCL/col-HA containing 10, 20, or 30% HA particles, and 3D-printed PCL-HA/col containing 10, 20, or 30% HA particles with collagen coating were fabricated. MC3T3-E1 pre-osteoblasts were cultured on the scaffolds for 14 days. The physicomechanical properties of the scaffolds and pre-osteoblast functionality were evaluated through experiments and finite element (FE) modeling. We found that coating of PCL scaffolds with collagen and HA or coating of PCL-HA composite scaffolds with collagen changed the geometry and topography of the scaffold surfaces. Furthermore, PCL/col-HA and PCL-HA/col showed higher surface roughness and elastic modulus, but lower water contact angle, than PCL scaffolds. FE-modeling showed that all scaffolds tolerated up to 2% compressive strain, which was lower than their yield stress. 3D-printed PCL/col-HA and PCL-HA/col scaffolds promoted pre-osteoblast proliferation and osteogenic activity compared to unmodified PCL scaffolds. PCL-HA/col scaffolds increased pre-osteoblast proliferation and collagen deposition, whereas PCL/col-HA scaffolds increased alkaline phosphatase activity and calcium deposition. Osteogenic activity of pre-osteoblasts was more enhanced on 3D-printed PCL/col-HA scaffolds than on PCL-HA/col scaffolds, particularly in the short-term, which seems promising for *in vivo* bone tissue engineering.

## Graphical abstract

**Graphical Abstract:**
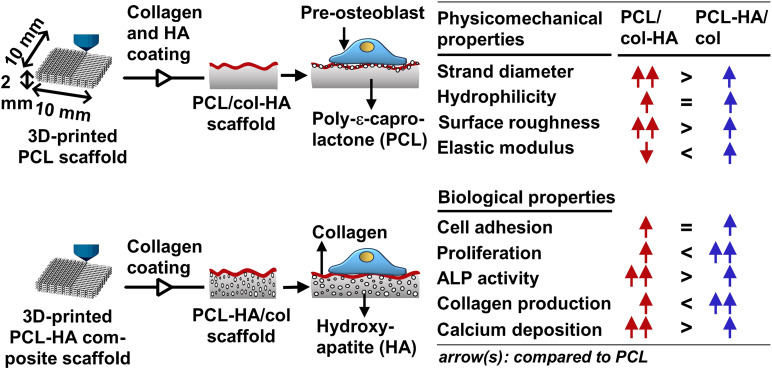

## Introduction

Bone tissue engineering uses three-dimensional (3D)-scaffolds to support bone regeneration. The ideal 3D-scaffold should have biomimetic physical and mechanical properties so that it can act as a temporary matrix to enhance cell bioactivity, *i.e*., cell adhesion, proliferation, and differentiation, and hence support tissue ingrowth.^1^ Biomimetic scaffold design is guided by the composition of the bone matrix, *e.g*., hydroxyapatite (HA) and collagen type I.^2^ The fabrication of a bioactive 3D-scaffold that can effectively mimic bone matrix architecture and composition is still a challenge. This requires not only the fabrication of a composite that mimicks the bone matrix, but also the creation of a 3D-scaffold with suitable physicomechanical and biological properties needed for bone tissue engineering.

Hydroxyapaptite (HA) is a bioactive material which is chemically similar to the mineral component of natural bone.^3^ It is widely used for bone repair in bone tissue engineering based on its osteoconductive property and bone bonding ability to surrounding tissue.^4^ 3D-scaffolds purely made of HA particles promote osteoblast attachment, proliferation, and differentiation.^5^ Unfortunately, these scaffolds are very brittle and cannot be used as implant material.^6^ Synthetic biopolymer composite scaffolds consisting of, *e.g*., poly-є-caprolactone (PCL) and HA particles can improve biocompatibility, hydrophilicity, and mechanical strength, as well as osteoblast attachment, proliferation, and differentiation.^1^ Ten to 30% HA was used based on prior studies demonstrating that this concentration range balances mechanical reinforcement (increasing elastic modulus without affecting brittleness) and bioactivity (promoting osteogenesis) in PCL-based scaffolds.^7^ HA concentrations below 10% offer insufficient bioactivity, while concentrations above 30% can compromise printability and flexibility.^8,9^ The 10%–30% HA concentration range has shown promise in preclinical models for load-bearing bone defects.^10^ Moreover, coating with HA particles on 3D-scaffolds is an alternative approach to increase the hydrophilicity, mechanical properties, and bioactivity of the scaffolds.^11^ The development of highly bioactive 3D-scaffolds containing HA particles to repair or replace damaged bone is still a challenge for bone tissue engineering.

In bone’s extracellular matrix (ECM), the most abundant protein is collagen type I.^12^ Based on biocompatibility and biodegradability of collagen, it has been utilized in creating scaffolds for bone tissue engineering.^13^ Usage of collagen type I in bone tissue engineering scaffolds provides a biomimetic environment for cells, which is beneficial for cell adhesion, spreading, migration, and proliferation.^13,14^ Unfortunately, the low mechanical strength of collagen limits its application in the field of bone tissue engineering.^15^ To improve the mechanical strength of collagen, it can be combined with bioactive inorganic materials, *e.g*., HA.^15^

3D-printing is an attractive fabrication process for usage in bone tissue engineering since it allows control of bulk geometry and the internal structure of scaffolds.^16,17^ PCL is a commonly used 3D-printable biopolymer.^18,19^ 3D-printed PCL scaffolds are valuable in terms of controllability, rapid prototyping, and high regular and interconnected porosity.^20^ While previous studies have explored collagen and/or hydroxyapatite functionalization of PCL scaffolds using various methods, *e.g*., wet-chemical or plasma treatment,^1,21,22^ only few studies have directly compared the effects of coating PCL scaffolds with collagen and HA (PCL/col-HA) versus incorporating HA into the PCL matrix and then coating with collagen (PCL-HA/col) on osteogenic activity.^23–25^ Our study fills this gap by providing a head-to-head comparison of these two strategies, revealing differential impact on pre-osteoblast proliferation, differentiation, and matrix deposition, which has implications for optimizing scaffold design in bone tissue engineering. In this study, we aimed to test whether 3D-printed PCL scaffolds with collagen-HA coating or PCL-HA composite scaffolds with collagen coating are more effective in stimulating osteogenic activity. Moreover, we characterized the scaffolds for their physicomechanical properties. 3D-printed PCL scaffolds with collagen-HA coating containing 10, 20, or 30% (wt/wt) HA particles, as well as 3D-printed PCL-HA composite scaffolds containing 10, 20, or 30% (wt/wt) HA particles with collagen coating were fabricated, and MC3T3-E1 pre-osteoblasts were cultured on the scaffolds for 14 days. The physicomechanical properties of the scaffolds, along with pre-osteoblast function, were analyzed by finite element (FE) modeling and experimental testing.

## Materials and methods

### 3D-printing of PCL and PCL/HA composite scaffolds

#### PCL/HA composite preparation

PCL (medical grade, MW 80 kDa; Sigma-Aldrich®, St. Louis, MO, USA) was dissolved in chloroform (Merck, Darmstadt, Germany) at 10% (wt/vol) by shaking for 2 h at 45°C. HA particles (phase purity 97.5%, particle size <50 µm; Apatech, Tehran, Iran) were dispersed in methanol (Merck, Darmstadt, Germany) at 10% (wt/vol) by shaking for 1 h at 60°C. The PCL and HA-containing solutions were mixed in a 1:1 (vol/vol) ratio. The solvent was evaporated at room temperature to produce dry PCL/HA composite sheets containing HA (0, 10, 20, or 30% (wt/wt)). The composite pellets were then stored at room temperature until used for 3D printing.

#### Scaffold design and fabrication

PCL or PCL/HA composite sheets with an HA concentration of 10, 20, and 30% (wt/wt) were 3D-printed layer-by-layer with an alternating 0°/90° lay-down pattern as described previously.^26^

### HA/collagen coating on 3D-printed PCL scaffolds

3D-printed PCL scaffolds underwent aminolysis using 10% (w/v) 1,6-hexanediamine (Merck, Darmstadt, Germany) in isopropanol (Merck) at 37°C for 1 h, followed by rinsing with deionized water. HA and collagen were coated layer-by-layer on the aminolysed PCL scaffolds using multilayer assembly. The aminolysed PCL scaffolds were HA-coated by immersion in a suspension containing HA particles at concentration of 10, 20, or 30% (wt/wt) for 15 min under gentle agitation, followed by rinsing with deionized water. The HA-coated scaffolds were collagen-coated by immersion in a solution containing 1 mg/ml collagen I (acid soluble collagen type I, bovine skin; Pasteur Institute, Tehran, Iran) in 0.01 M hydrochloric acid (HCL; Merck, Darmstadt, Germany) for another 15 min at 4°C. This alternate coating process was repeated until twenty layers were obtained. All experiments were conducted using collagen I from the same batch to exclude potential batch variability.

### Collagen coating on 3D-printed PCL/HA composite scaffolds

3D-printed PCL/HA composite scaffolds containing 10, 20, or 30% (wt/wt) HA particles were aminolysed as described above (section 2.2). The aminolysed composite scaffolds were collagen coated by immersion in a solultion containing 1 mg/ml collagen (acid soluble collagen type I, bovine skin; Pasteur Institute) in 0.01 M hydrochloric acid (HCL; Merck) for 24 h at 4°C. All experiments were conducted using collagen I from the same batch to exclude potential batch variability.

### Scaffold characterization

#### Crystalline phases

The crystalline phases of HA particles within the composite structure of the PCL scaffolds, either containing or coated with 10, 20, or 30% (wt/wt) HA particles, were identified using X-ray diffraction (XRD) analysis as described previously.^1^

#### Strand diameter and void size

Ten strands and voids were measured for each scaffold by ImageJ (https://imagej.net/downloads). Scaffolds were analyzed in triplicate.

#### Surface topography and morphology

The surface topography and morphology of the 3D-printed PCL, PCL/col-HA, and PCL-HA/col scaffolds were examined using scanning electron microscopy (SEM) as described.^20,26^

#### Hydrophilicity

To avoid possible effects of scaffold curvature and roughness on the hydrophilicity of PCL/col-HA and PCL-HA/col scaffolds, flat PCL surfaces were prepared by solvent casting, and coated with HA-collagen containing 10, 20, or 30% HA particles. Flat PCL/HA composite surfaces containing 10, 20, or 30% HA particles were prepared by solvent casting, and coated with collagen. A video contact angle system (Sony color video camera, Tokyo, Japan) was used to capture the water contact angle, which was quantified using ImageJ software. Each sample was analyzed in three independent replicates.

#### Surface roughness

Non-contact optical profilometer (Fanavari Kahroba Co., Tehran, Iran) was utilized to detect the surface roughness of scaffolds as described previously.^20^ Measurements were performed in three independent replicates.

#### Mechanical properties

To evaluate the compressive strength of the scaffolds, an STM 20 universal testing machine (Santam, Tehran, Iran) equipped with a 200 N load cell was utilized at a rate of 1 mm/min at room temperature (25°C), as described earlier.^20^ Scaffolds were tested in three independent replicates.

### FE modeling

A 3D-printed scaffold measuring 10 × 10 × 2 mm (length × width × height) with strands of 0.4 mm diameter and void size of 0.3 mm, was created using FE modeling software (COMSOL Multiphysics 5.4, Stockholm, Sweden). The mechanical behavior of the PCL, PCL/col-HA, and PCL-HA/col scaffolds under uniform 2% compression strain was simulated through FE analysis using COMSOL Multiphysics 5.4 as previously described..^20^ The models consisted of a mesh comprising 55,091 tetrahedra, 31,516 triangles, 7848 edges, and 1300 vertex elements. Parameters and default values used in the FE modeling are provided in Table 1.

**Table 1.** FE modeling: parameters and default values used.

Type of scaffold	Parameter	Expression	Value	Reference
PCL	E (MPa)	Elastic modulus	90.4	
	ϑ	Poisson’s ratio	0.45	^16^
	ρ (gr/ml)	Density	1.145	
PCL/col-HA10%	E (MPa)	Elastic modulus	52.6	
	ϑ	Poisson’s ratio	0.45	
	ρ (gr/ml)	Density	1.145	
PCL/col-HA20%	E (MPa)	Elastic modulus	91.7	
	ϑ	Poisson’s ratio	0.45	
	ρ (gr/ml)	Density	1.145	
PCL/col-HA30%	E (MPa)	Elastic modulus	112.1	
	ϑ	Poisson’s ratio	0.45	
	ρ (gr/ml)	Density	1.145	
PCL-HA10%/col	E (MPa)	Elastic modulus	122.2	
	ϑ	Poisson’s ratio	0.45	
	ρ (gr/ml)	Density	1.145	
PCL-HA20%/col	E (MPa)	Elastic modulus	141.6	
	ϑ	Poisson’s ratio	0.45	
	ρ (gr/ml)	Density	1.145	
PCL-HA30%/col	E (MPa)	Elastic modulus	144.1	
	ϑ	Poisson’s ratio	0.45	
	ρ (gr/ml)	Density	1.145	

PCL, poly-ε-caprolactone; col, collagen; HA, hydroxyapatite.

### Cell culture and bioactivity

#### Cell culture and seeding

Mouse embryonic pre-osteoblasts (MC3T3-E1; American Type Culture Collection, Manassas, VA, USA) were cultured in α-Minimum Essential Medium (α-MEM; Gibco, Life Technologies), supplemented with 10% fetal bovine serum (FBS; Gibco), 1% PSF antibiotic-antimycotic solution (Sigma-Aldrich®, St. Louis, MO), 50 µg/ml ascorbic acid (Sigma-Aldrich®), and 10 mM β-glycerophosphate (Sigma-Aldrich®) at 37°C in humidified air containing 5% CO_2_ as described earlier.^20^ The cells were tested for mycoplasma contamination using polymerase chain reaction (PCR) analysis and the results confirmed the cells were free of contamination for a period of 4 months. Cells were seeded on the scaffolds by spreading three 10 µl drops of a 5 × 10^5^ cells/cm^3^ cell suspension onto the surface of the scaffold (l × w × h:5 × 5 × 2 mm; volume: 50 mm^3^) in 48-well culture plates as described previously.^20^ Following cell seeding, cells were allowed to diffuse and attach throughout the scaffolds for 8 h. Cell-seeded scaffolds were washed twice with PBS before being transferred to a new 48 well culture plate. Seeding efficiency was determined by quantification of the number of cells attached to the culture plate and on the scaffolds using the AlamarBlue® fluorescent assay (Invitrogen). Scaffolds were cultured up to 14 days. Three independent experiments with three constructs per group were performed.^20^

#### Cell morphology and spreading

After 14 days of culture, SEM was used to visualize cell morphology and spreading on all scaffold types. Cell-seeded scaffolds were fixed using 4% glutaraldehyde, dehydrated in graded ethanol series, and coated with gold using an Edwards Sputter Coater S150B. Imaging was performed using a Zeiss EVO LS-15 scanning electron microscope at an accelerating voltage of 20 kV.

#### Alkaline phosphatase activity

To assess osteoblastic differentiation, alkaline phosphatase (ALP) activity was measured in cell-seeded scaffolds after 14 days of culture. ALP activity was normalized to the total cellular protein content determined using a BCA Protein Assay Kit (Pierce^TM^, Rockford, Ill, USA). Cell-seeded scaffolds were assayed in three independent biological replicates.

#### Cell proliferation

Cell proliferation on the scaffolds was quantified at days 3, 7, and 14 using AlamarBlue® fluorescent assay. Values were normalized to the cell number in the scaffolds at day one.^26^ Data was obtained from three cell-seeded scaffolds from three independent experiments (n = 3).

#### Collagen production

After 14 days of culture, cell-seeded scaffolds were washed with PBS, and fixed in 4% formaldehyde. Collagen production by pre-osteoblasts was visualized and quantified using a picrosirius red staining kit (Chondrex, Inc., Redmond, WA, USA), as described earlier.^20^ All experiments were repeated in three independent biological replicates.

#### Calcium deposition

Calcium deposition was visualized and quantified using Alizarin Red staining (Merck, Darmstadt, Germany) after 14 days of culture, as described earlier.^20^ Calcium deposition was expressed as absorbance (450 nm) per gram scaffold. All experiments were repeated in three independent biological replicates.

### Statistical analysis

Data are mean ± standard deviation (SD). Statistical analysis was conducted using GraphPad Prism software (GraphPad Software Ink., San Diego, CA, USA). Differences in mean values were assessed via two-way ANOVA followed by Bonferroni post-hoc tests. A p-value <0.05 was considered statistically significant.

## Results

### Crystalline phase identification

**Figure 1.**
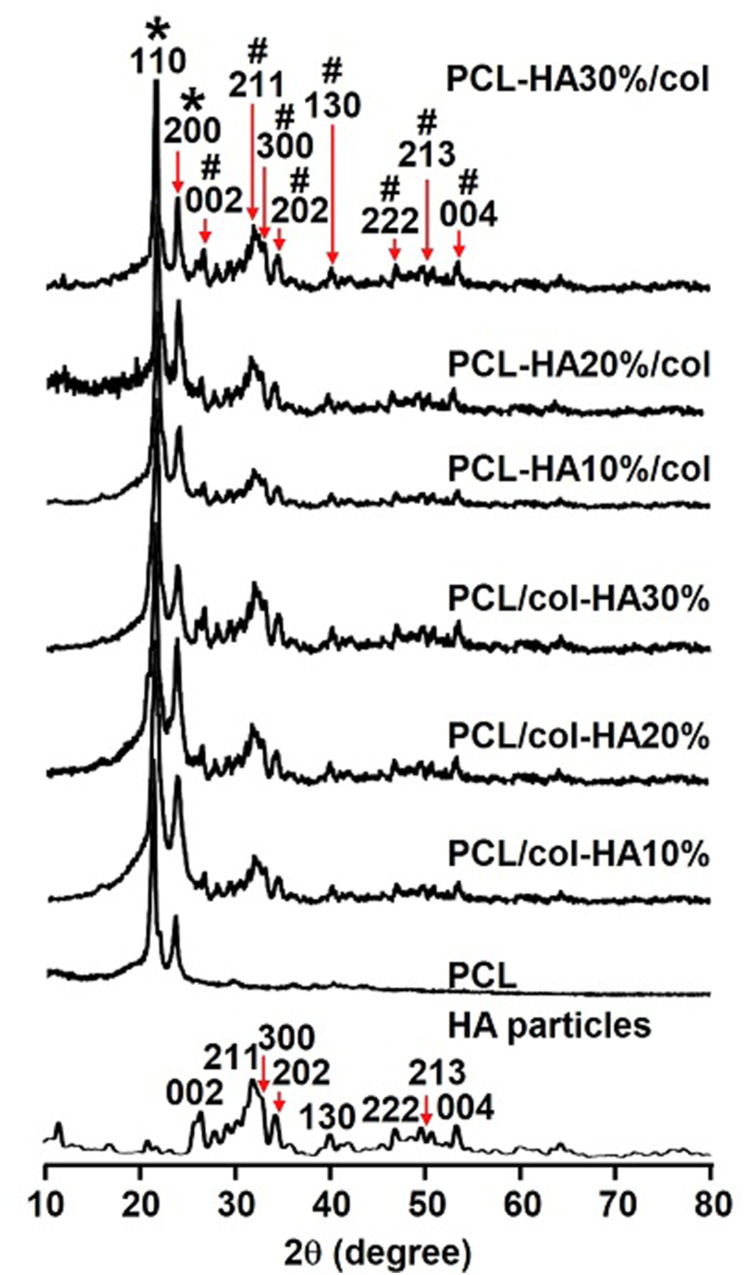
Crystalline phase identification of 3D-printed PCL scaffolds without or with collagen-HA coating as well as PCL-HA composite scaffolds with collagen coating determined by X-ray powder diffraction (XRD) analysis. PCL/col-HA scaffolds contained 10, 20, or 30% (wt/wt) HA in collagen. PCL-HA/col contained 10, 20, or 30% (wt/wt) HA in PCL. The peaks at (110) and (200) corresponded to the planes of the orthorhombic crystal structure of PCL. The peaks at (002), (211), (300), (202), (130), (222), (213), and (004) corresponded to the planes of the crystal structure of HA particles. XRD, X-ray powder diffraction; PCL, poly-є-caprolactone; HA, hydroxyapatite; col, collagen, PCL/col-HA, PCL scaffold with collagen-HA coating; PCL-HA/col, PCL-HA composite scaffold with collagen coating.

X-ray powder diffraction (XRD) analysis was used to identify crystalline phases in the PCL-based scaffolds with or without collagen-HA coating as well as in PCL-HA composite scaffolds coated with collagen (Figure 1). Phase identification of HA particles, pure PCL, PCL/col-HA10%, PCL/col-HA20%, PCL/col-HA30%, PCL-HA10%/col, PCL-HA20%/col, and PCL-HA30%/col were analyzed (Figure 1). XRD peaks corresponding to the (110) and (200) planes of the orthorhombic crystal structure of PCL were observed at angles 21.15° and 24.51° (Figure 1).^27,28^ Moreover, the peaks at 26.91°, 32.65°, 33.35°, 40.24°, 47.12°, 49.95°, and 53.61 corresponded to the (002), (211), (202), (130), (222), (213), and (004), planes of the crystal structure of HA particles (according to JCPDS09-0432) (Figure 1).^29,30^ With increasing HA concentration in both PCL-HA/col and PCL/col-HA scaffolds, the intensity of the crystallinity peaks associated with HA’s crystal structure also increased (Figure 1).

### Strand diameter and void size

**Figure 2.**
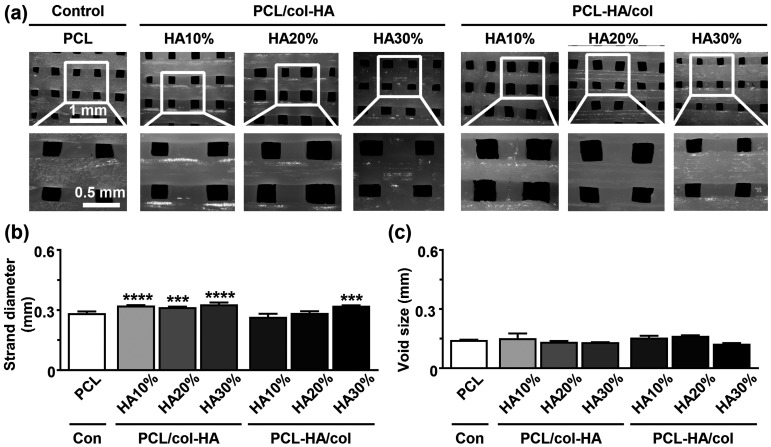
Strand diameter and void size in 3D-printed PCL scaffolds without or with collagen-HA coating as well as PCL-HA composite scaffolds with collagen coating. PCL/col-HA scaffolds contained 10, 20, or 30% (wt/wt) HA in collagen. PCL-HA/col composite scaffolds contained 10, 20, or 30% (wt/wt) HA in PCL. (a) Top view of the scaffolds revealing the internal structure, including strands and voids. (b) Strand diameter in the scaffolds. (c) Void size in the scaffolds. PCL, poly-є-caprolactone; HA, hydroxyapatite; col, collagen, PCL/col-HA, PCL scaffold with collagen-HA coating; PCL-HA/col, PCL-HA composite scaffold with collagen coating. Values are mean ± SD (n = 3). ***Significantly different from unfunctionalized 3D-printed PCL scaffolds, *p* < 0.0005, ****p < 0.0001.

All scaffolds exhibited a regular structure with interconnected pores (Figure 2(a)). The strands diameter in the scaffolds was 0.29 ± 0.008 mm (mean ± SD) for PCL, 0.32 ± 0.002 mm for PCL/col-HA10%, 0.31 ± 0.003 mm for PCL/col-HA20%, 0.33 ± 0.009 mm for PCL/col-HA30%, 0.27 ± 0.015 mm for PCL-HA10%/col, 0.29 ± 0.009 mm for PCL-HA20%/col, and 0.32 ± 0.002 mm for PCL-HA30%/col (Figure 2(b)). The strand diameter was significantly higher in PCL/col-HA10% (1.13-fold increase, *p* < 0.0001), PCL/col-HA20% (1.11-fold increase, *p* < 0.0005), PCL/col-HA30% (1.15-fold increase, *p* < 0.0001), and PCL-HA30%/col (1.13-fold increase, *p* < 0.0005) than in 3D-printed PCL scaffolds. The void size of the scaffolds was 0.14 ± 0.001 mm (mean ± SD) for PCL, 0.15 ± 0.025 mm for PCL/col-HA10%, 0.13 ± 0.004 mm for PCL/col-HA20%, 0.13 ± 0.001 mm for PCL/col-HA30%, 0.15 ± 0.010 mm for PCL-HA10%/col, 0.16 ± 0.003 mm for PCL-HA20%/col, and 0.12 ± 0.003 mm for PCL-HA30%/col (Figure 2(c)). The strand diameter in the 3D-printed scaffolds enhanced with increasing HA concentration in the scaffolds, while the void size of the scaffolds decreased simultaneously.

### Surface topography

**Figure 3.**
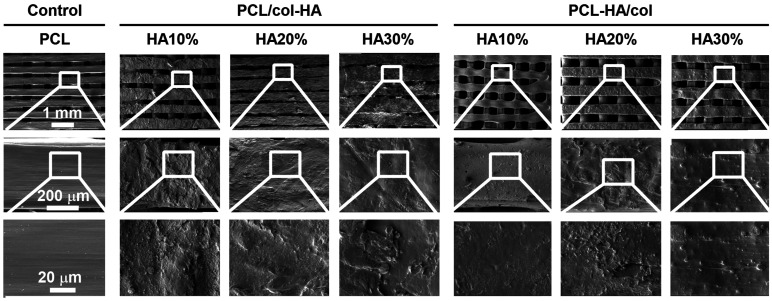
SEM images of 3D-printed PCL, PCL-HA/col, and PCL/col-HA scaffolds containing 10, 20, or 30% (wt/wt) HA. First row: scaffold structure; second row: strand topography; third row: scaffold surface topography. Note: Images were taken at different magnification. SEM, scanning electron microscopy; PCL, poly-є-caprolactone; HA, hydroxyapatite; PCL-HA/col, PCL-HA composite scaffold with collagen coating; PCL/col-HA, PCL scaffold without or with collagen-HA coating.

Surface topography of 3D-printed PCL scaffolds without or with collagen-HA coating as well as PCL-HA composite scaffolds with collagen coating was determined on SEM images (Figure 3). Unmodified PCL scaffolds displayed a regular structure and smooth surface (Figure 3). Coating PCL scaffolds with collagen and HA or coating PCL-HA composite scaffolds with collagen changed the geometry, shape, and topography of the 3D-printed PCL scaffold surfaces (Figure 3). PCL/col-HA and PCL-HA/col scaffolds showed a ruffled surface pattern visible at higher magnifications (Figure 3). Surface roughness of both PCL/col-HA and PCL-HA/col scaffolds enhanced by increasing HA concentration (Figure 3).

### Surface hydrophilicity

**Figure 4.**
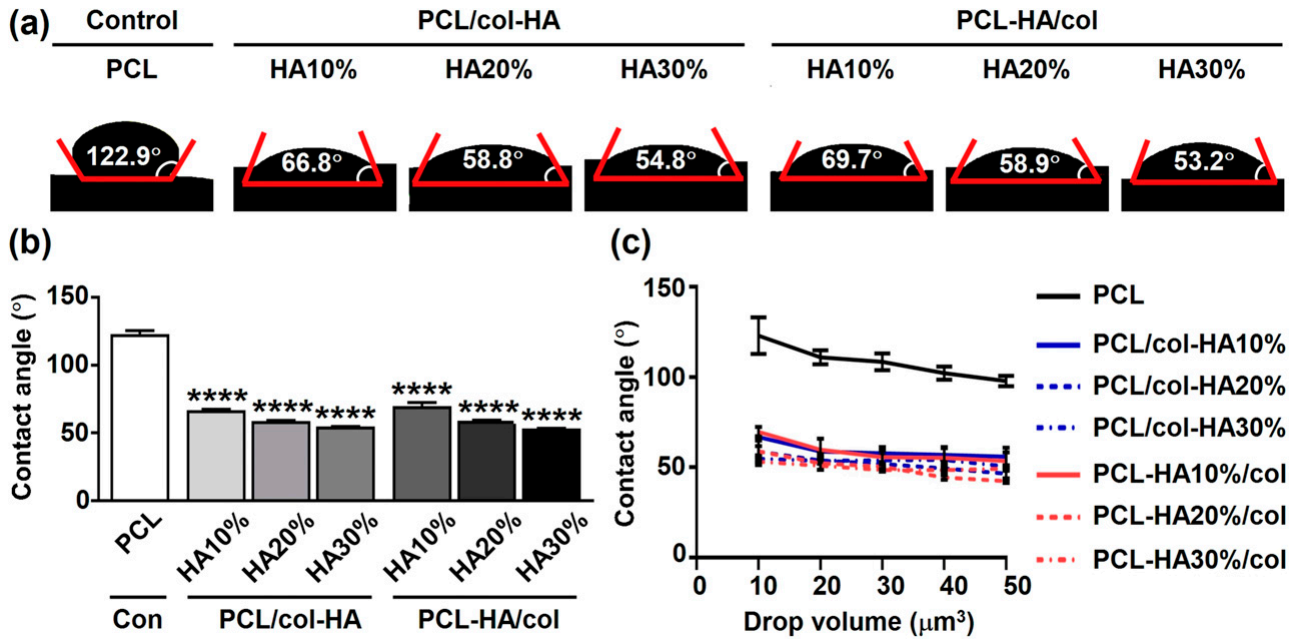
Surface hydrophilicity of 3D-printed PCL scaffolds without or with collagen-HA coating as well as PCL-HA composite scaffolds with collagen coating. PCL/col-HA scaffolds contained 10, 20, or 30% (wt/wt) HA in collagen. PCL-HA/col composite scaffolds contained 10, 20, or 30% (wt/wt) HA in PCL. (a) Water contact angle on scaffold surface. Red line: contact angle. (b) Average water contact angle. (c) Drop size dependence of scaffold surface hydrophilicity. PCL, poly-є-caprolactone; HA, hydroxyapatite; col, collagen; PCL/col-HA, PCL scaffold with collagen-HA coating; PCL-HA/col, PCL-HA composite scaffold with collagen coating. Values are mean ± SD (n = 3). ****Significantly different from 3D-printed PCL scaffolds, *p* < 0.0001.

Surface hydrophilicity of the scaffolds was assessed through water contact angle measurement (Figure 4(a)). The water contact angle of the scaffolds was 122.9 ± 2.47° (mean ± SD) for PCL, 66.8 ± 0.60° for PCL/col-HA10%, 58.83 ± 0.64° for PCL/col-HA20%, 54.77 ± 0.06° for PCL/col-HA30%, 69.73 ± 2.75° for PCL-HA10%/col, 58.93 ± 0.55° for PCL-HA20%/col, and 53.23 ± 0.23° for PCL-HA30%/col (Figure 4(b)). The water contact angle of 3D-printed PCL scaffolds decreased significantly by increasing the HA concentration (PCL/col-HA10%: 0.54-fold decrease, *p* < 0.0001; PCL/col-HA20%: 0.48-fold decrease, *p* < 0.0001; PCL/col-HA30%: 0.45-fold decrease, *p* < 0.0001; PCL-HA10%/col: 0.57-fold decrease, *p* < 0.0001; PCL-HA20%/col: 0.48-fold decrease, *p* < 0.0001; PCL-HA30%/col: 0.43-fold decrease, *p* < 0.0001) compared to PCL scaffolds (Figure 4(b)). By increasing the water drop volume from 10 to 50 μm^3^, the water contact angle decreased (PCL: 0.80; PCL/col-HA10%: 0.84; PCL/col-HA20%: 0.79; PCL/col-HA30%: 0.92; PCL-HA10%/col: 0.77; PCL-HA20%/col: 0.72; PCL-HA30%/col: 0.93; Figure 4(c)).

### Surface roughness

**Figure 5.**
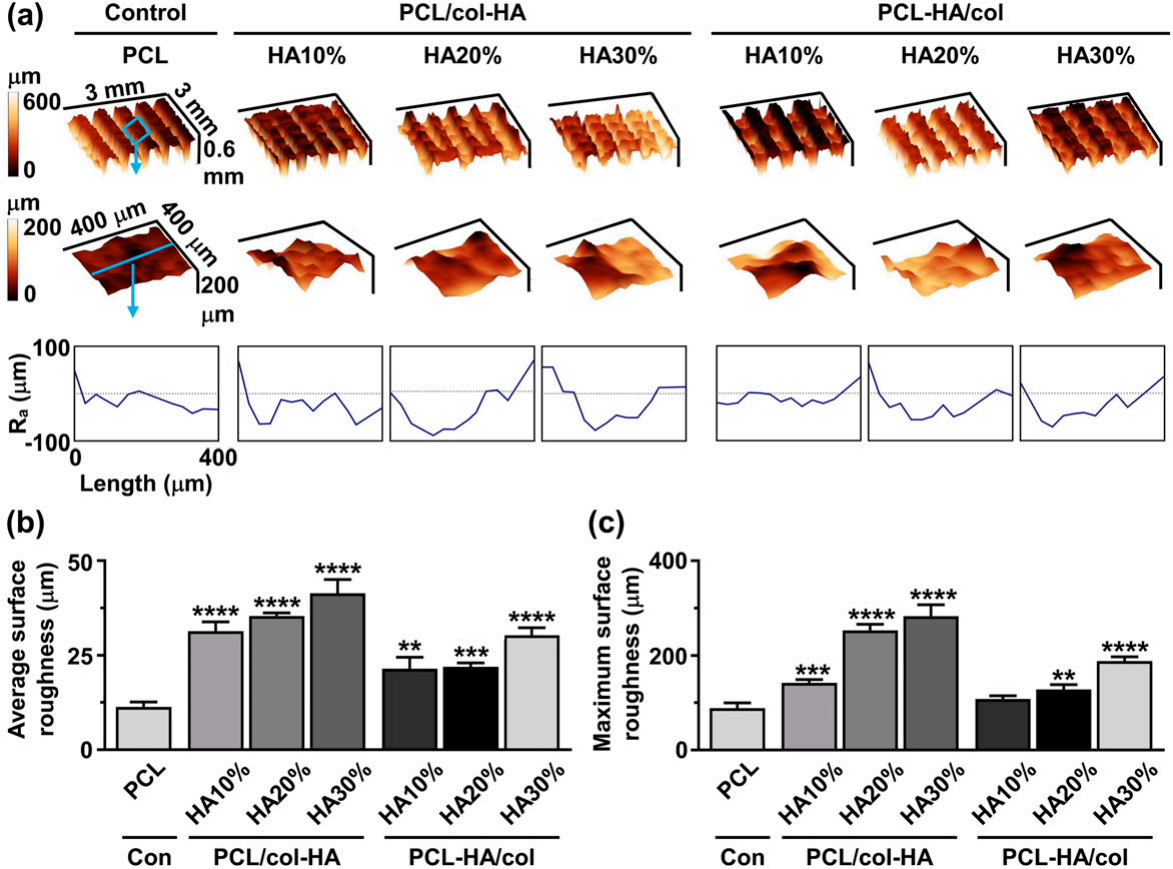
Surface roughness magnitude and distribution in 3D-printed PCL scaffolds without or with collagen-HA coating as well as PCL-HA composite scaffolds with collagen coating. PCL/col-HA scaffolds contained 10, 20, or 30% (wt/wt) HA in collagen. PCL-HA/col composite scaffolds contained 10, 20, or 30% (wt/wt) HA in PCL. (a) 3D- and 2D-View of surface roughness distribution and magnitude on scaffold surface. Ra, average surface roughness. (b) Average scaffold surface roughness. (c) Maximum scaffold surface roughness. PCL, poly-є-caprolactone; HA, hydroxyapatite; col, collagen; PCL/col-HA, PCL scaffold with collagen-HA coating; PCL-HA/col, PCL-HA composite scaffold with collagen coating. Values are mean ± SD (n = 3). **Significantly different from 3D-printed PCL scaffolds, *p* < 0.005, ***p < 0.0005, ****p < 0.0001.

Surface roughness of 3D-printed PCL scaffolds without or with collagen-HA coating as well as PCL-HA composite scaffolds with collagen coating was determined by surface profilometry (Figure 5). 3D profilometry revealed variations in the distribution and magnitude of surface roughness across the scaffolds (Figure 5(a)). The average surface roughness of the strands of 3D-printed scaffolds was 11.30 ± 1.35 µm (mean ± SD) for PCL, 31.33 ± 2.47 µm for PCL/col-HA10%, 35.41 ± 0.81 µm for PCL/col-HA20%, 41.33 ± 3.68 µm for PCL/col-HA30%, 21.43 ± 3.04 µm for PCL-HA10%/col, 21.93 ± 1.05 µm for PCL-HA20%/col, and 30.31 ± 1.97 µm for PCL-HA30%/col (Figure 5(b)). The average surface roughness of 3D-printed PCL scaffolds showed a significant increase with higher HA concentrations. Specifically, PCL/col-HA10% exhibited a 2.77-fold increase (*p* < 0.0001), PCL/col-HA20% a 3.13-fold increase (*p* < 0.0001), PCL/col-HA30% a 3.66-fold increase (*p* < 0.0001), PCL-HA10%/col a 1.90-fold increase (*p* < 0.005), PCL-HA20%/col a 1.94-fold increase (*p* < 0.0005), and PCL-HA30%/col a 2.68-fold increase (*p* < 0.0001) compared to unmodified PCL scaffolds (Figure 5(b)). The maximum surface roughness of the strands of 3D-printed scaffolds was 88.70 ± 11.00 µm (mean ± SD) for PCL, 142.00 ± 7.11 µm for PCL/col-HA10%, 252.93 ± 12.92 µm for PCL/col-HA20%, 283.30 ± 24.00 µm for PCL/col-HA30%, 108.00 ± 6.95 µm for PCL-HA10%/col, 128.23 ± 10.43 µm for PCL-HA20%/col, and 188.60 ± 8.59 µm for PCL-HA30%/col (Figure 5(c)). The maximum surface roughness of 3D-printed PCL scaffolds increased significantly by increasing the HA concentration (PCL/col-HA10%: 1.60-fold increase, *p* < 0.0005; PCL/col-HA20%: 2.85-fold increase, *p* < 0.0001; PCL/col-HA30%: 3.19-fold increase, *p* < 0.0001; PCL-HA20%/col: 1.45-fold increase, *p* < 0.005; PCL-HA30%/col: 2.13-fold increase, *p* < 0.0001) compared to PCL scaffolds (Figure 5(b)). The PCL/col-HA30% scaffolds had the highest average and maximum surface roughness compared to all other scaffolds (Figure 5(b), and (c)).

### Mechanical characteristics of 3D-printed scaffolds

**Figure 6.**
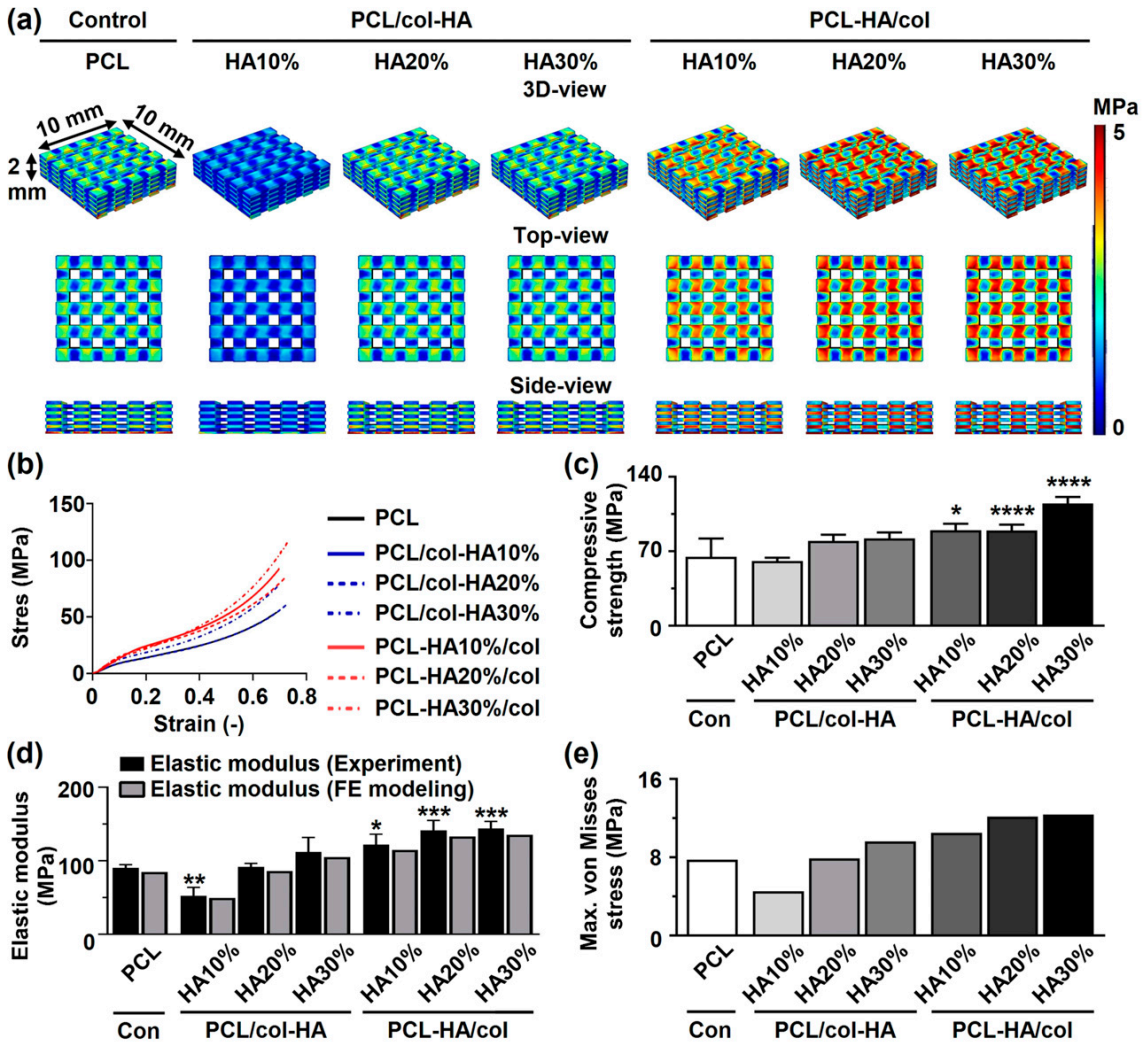
Mechanical characteristics of 3D-printed PCL scaffolds without or with collagen-HA coating as well as PCL-HA composite scaffolds with collagen coating. PCL/col-HA scaffolds contained 10, 20, or 30% (wt/wt) HA in collagen. PCL-HA/col composite scaffolds contained 10, 20, or 30% (wt/wt) HA in PCL. (a) 3D-, top-, and side-views of von Mises stress distribution in the scaffolds, resulting from uniform 2% compressive strain, as determined by FE modeling. (b) Compressive strength of the scaffolds. (c) Elastic modulus of the scaffolds determined experimentally and by FE-modeling. (d) Maximum von Mises stress of the scaffolds. PCL, poly-є-caprolactone; HA, hydroxyapatite; col, collagen; PCL/col-HA, PCL scaffold with collagen-HA coating; PCL-HA/col, PCL-HA composite scaffold with collagen coating. Values are mean ± SD (n = 3). *Significantly different from 3D-printed PCL scaffolds, *p* < 0.05, **p < 0.005, ***p < 0.0005, ****p < 0.0001.

The mechanical properties of 3D-printed PCL scaffolds without or with collagen-HA coating as well as PCL-HA composite scaffolds with collagen coating were determined by experiments and FE-modeling (Figure 6). The von Mises stress distribution on PCL/col-HA10% scaffolds was less uniform compared to other scaffold types (Figure 6(a)). Coating of PCL scaffolds with collagen and HA or coating PCL-HA composite scaffolds with collagen did not alter the stress-strain relationship of the PCL scaffolds (Figure 6(b)). The compressive strength was significantly higher for PCL-HA10%/col (1.39-fold increase, *p* < 0.05), PCL-HA20%/col: (1.38-fold increase, *p* < 0.0001), and PCL-HA30%/col (1.78-fold increase, *p* < 0.0001) scaffolds, but not PCL/col-HA10%, PCL/col-HA20%, and PCL/col-HA30% scaffolds, than for 3D-printed PCL scaffolds (64.75 ± 17.19 MPa (mean ± SD); Figure 6(c)). The elastic modulus was significantly lower for PCL/col-HA10% scaffolds (0.58-fold, *p* < 0.005), but significantly higher for PCL-HA10%/col (1.35-fold increase, *p* < 0.05), PCL-HA20%/col: (1.56-fold increase, *p* < 0.0005), and PCL-HA30%/col: (1.59-fold increase, *p* < 0.0005) scaffolds compared to PCL scaffolds (90.43 ± 4.41 MPa (mean ± SD); Figure 6(d)). Comparison between FE modeling and experimental results showed a variation in mean values that fell within an acceptable range for validating FE modeling with experimental data (Figure 6(d)). Under 2% compression strain, the maximum von Mises stress for all scaffold types ranged from 4.51 MPa to 12.36 MPa (Figure 6(e)). Von Mises stress distribution varied among scaffold types but remained below the yield stress of bulk materials under compression strain (Figure 6(e)).

### Pre-osteoblast bioactivity

**Figure 7.**
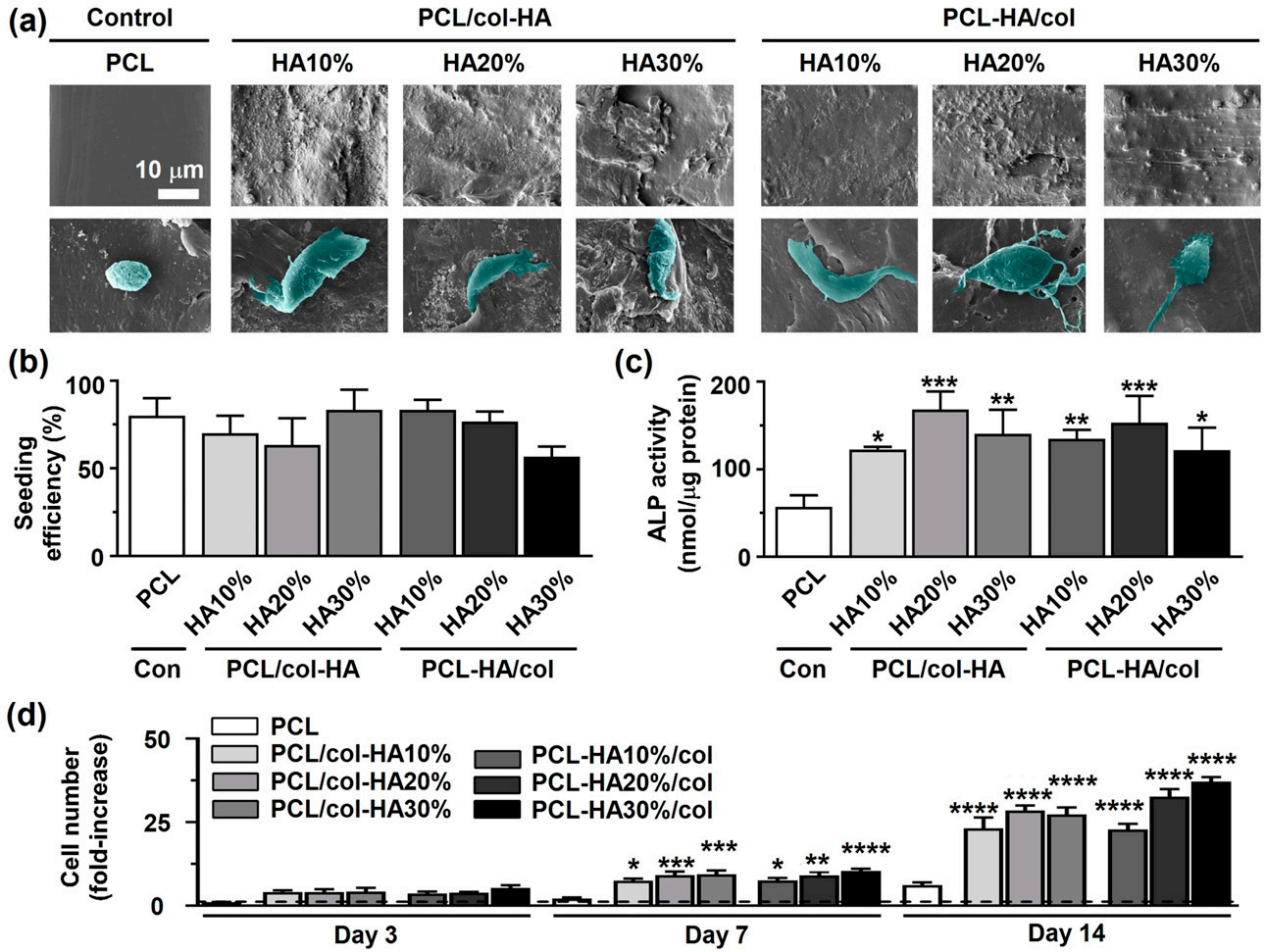
Pre-osteoblast bioactivity in 3D-printed PCL scaffolds without or with collagen-HA coating as well as PCL-HA composite scaffolds with collagen coating. PCL/col-HA scaffolds contained 10, 20, or 30% (wt/wt) HA in collagen. PCL-HA/col composite scaffolds contained 10, 20, or 30% (wt/wt) HA in PCL. (a) Pre-osteoblast morphology on the scaffolds. (b) Cell seeding efficiency on the scaffolds. (c) ALP activity of pre-osteoblasts in the scaffolds after 14 days (d) Pre-osteoblast proliferation on the scaffolds after 3, 7, and 14 days. PCL, poly-є-caprolactone; HA, hydroxyapatite; col, collagen; PCL/col-HA, PCL scaffold with collagen-HA coating; PCL-HA/col, PCL-HA composite scaffold with collagen coating. Values are mean ± SD (n = 3). *Significantly different from 3D-printed PCL scaffolds, *p* < 0.05, **p < 0.005, ***p < 0.0005, ****p < 0.0001.

Pre-osteoblast morphology on 3D-printed PCL scaffolds without or with collagen-HA coating as well as PCL-HA composite scaffolds with collagen coating by after 14 days of culture was determined on SEM images (Figure 7(a)). Pre-osteoblasts had slightly spherical morphology on the surface of 3D-printed PCL scaffolds (Figure 7(a)). Pre-osteoblasts spread more on both 3D-printed PCL/col-HA scaffolds, containing 10, 20, or 30% (wt/wt) HA in collagen, and PCL-HA/col, containing 10, 20, or 30% (wt/wt) HA in PCL, compared to 3D-printed PCL scaffolds. The seeding efficiency ranged from 57% (on PCL-HA30%/col scaffolds) to 83% (on PCL/col-HA30% and PCL-HA10%/col scaffolds) (Figure 7(b)). The ALP activity was significantly higher on PCL/col-HA10% (2.15-fold increase, *p* < 0.05), PCL/col-HA20% (2.95-fold increase, *p* < 0.0005), and PCL/col-HA30% (2.46-fold increase, *p* < 0.005), PCL-HA10%/col (2.36-fold increase, *p* < 0.005), PCL-HA20%/col: (2.69-fold increase, *p* < 0.0005), and PCL-HA30%/col (2.14-fold increase, *p* < 0.05) scaffolds than on 3D-printed PCL scaffolds (57.01 ± 13.24 nmol/µg protein (mean ± SD); Figure 7(c)). Pre-osteoblasts proliferated more on both 3D-printed PCL/col-HA scaffolds, containing 10, 20, or 30% (wt/wt) HA in collagen, and PCL-HA/col, containing 10, 20, or 30% (wt/wt) HA in PCL, than on PCL scaffolds after 3, 7, and 14 days in relation to day 1 (Figure 7(d)). At day 3, proliferation was similar on all types of scaffolds, but at day 7 it was significantly higher on PCL/col-HA10% (3.48-fold increase, *p* < 0.05), PCL/col-HA20% (4.26-fold increase, *p* < 0.0005), PCL/col-HA30% (4.35-fold increase, *p* < 0.0005), PCL-HA10%/col (3.49-fold increase, *p* < 0.05), PCL-HA20%/col (4.19-fold increase, *p* < 0.005), and PCL-HA30%/col (4.82-fold increase, *p* < 0.0001) scaffolds than on PCL scaffolds (2.15 ± 0.29 (mean ± SD); Figure 7(d)). At day 14, proliferation was significantly higher on PCL/col-HA10% (3.78-fold increase, *p* < 0.0001), PCL/col-HA20% (4.65-fold increase, *p* < 0.0001), PCL/col-HA30% (4.47-fold increase, *p* < 0.0001), PCL-HA10%/col (3.73-fold increase, *p* < 0.0001), PCL-HA20%/col: (5.33-fold increase, *p* < 0.0001), and PCL-HA30%/col (6.07-fold increase, *p* < 0.0001) scaffolds than on PCL scaffolds (6.11 ± 0.82 (mean ± SD); Figure 7(d)).

### Collagen matrix and calcium deposition

**Figure 8.**
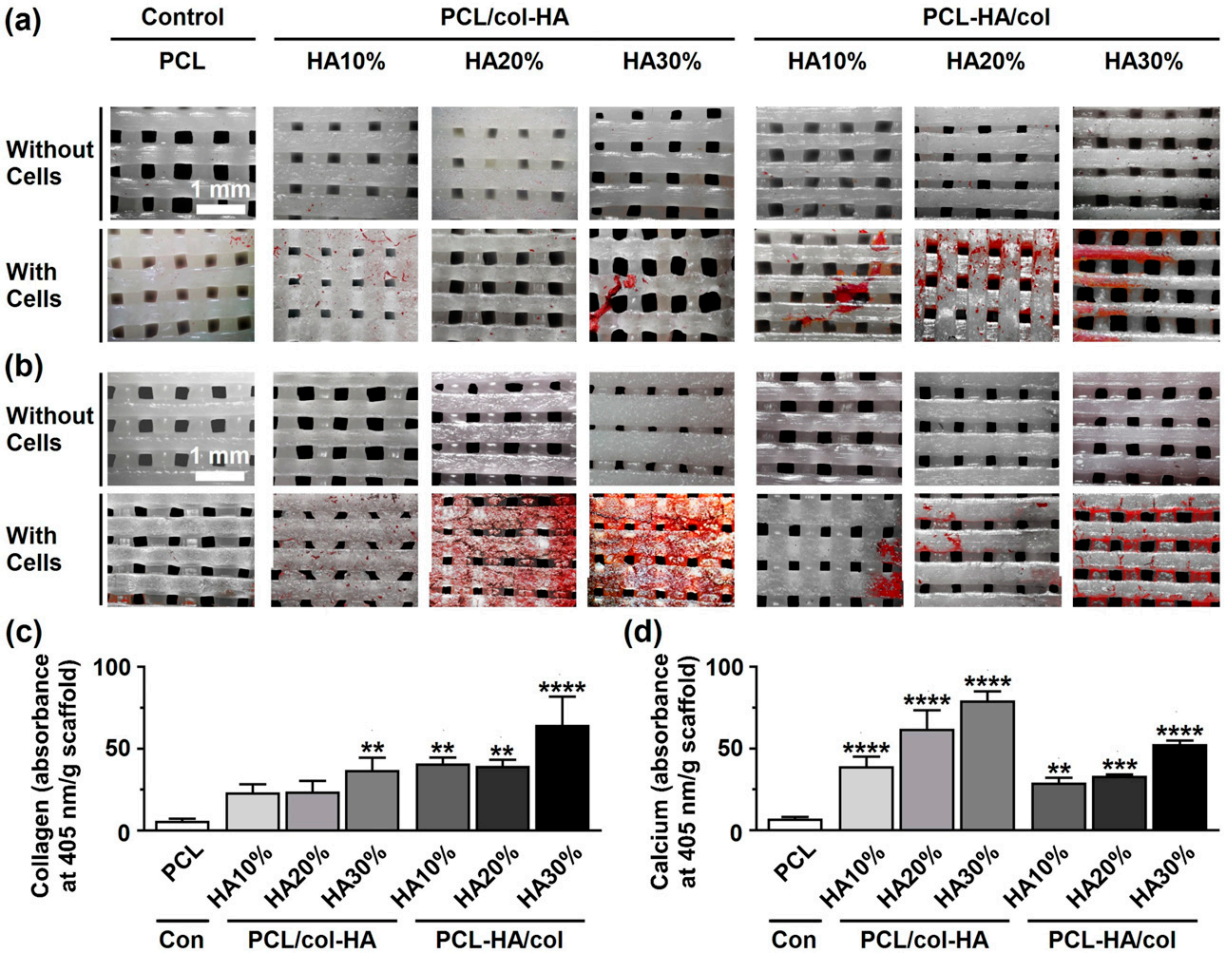
Collagen matrix and calcium deposition by pre-osteoblasts on 3D-printed PCL scaffolds without or with collagen-HA coating as well as PCL-HA composite scaffolds with collagen coating after 14 days of culture. PCL/col-HA scaffolds contained 10, 20, or 30% (wt/wt) HA in collagen. PCL-HA/col composite scaffolds contained 10, 20, or 30% (wt/wt) HA in PCL. (a) Collagenous matrix deposition (picrosirius red staining). (b) Calcium deposition (alizarin red staining). (c) Quantification of collagen deposition. (d) Quantification of calcium content. PCL, poly-є-caprolactone; HA, hydroxyapatite; col, collagen; PCL/col-HA, PCL scaffold with collagen-HA coating; PCL-HA/col, PCL-HA composite scaffold with collagen coating. Values are mean ± SD (n = 3). **Significantly different from 3D-printed PCL scaffolds, *p* < 0.005, ***p < 0.0005, ****p < 0.0001.

Collagen matrix formation and calcium deposition by pre-osteoblasts on 3D-printed PCL scaffolds with or without collagen-HA coating, as well as on PCL-HA composite scaffolds coated with collagen were evaluated (Figure 8). Among all scaffolds, PCL-HA30%/col demonstrated the highest collagen deposition after 14 days, evidenced by more intense red staining (Figure 8(a)). Calcium deposition (red) was highest, *i.e*. more intense red staining, on PCL/col-HA30% scaffolds after 14 days compared to all other scaffolds (Figure 8(b)). At day 14, collagen production was higher on PCL/col-HA30% (6.15-fold increase, *p* < 0.005), PCL-HA10%/col (6.80-fold increase, *p* < 0.005), PCL-HA20%/col (6.54-fold increase, *p* < 0.005), and PCL-HA30%/col (10.69-fold increase, *p* < 0.0001) scaffolds, but not on PCL/col-HA10% and PCL/col-HA20% scaffolds, than on PCL scaffolds (6.04 ± 1.21 absorbance at 405 nm/g scaffold (mean ± SD); Figure 8(c)). At day 14, calcium deposition was higher on PCL/col-HA10% (5.50-fold increase, *p* < 0.0001), PCL/col-HA20% (8.72-fold increase, *p* < 0.0001), PCL/col-HA30% (11.14-fold increase, *p* < 0.0001), PCL-HA10%/col (4.10-fold increase, *p* < 0.005), PCL-HA20%/col (4.67-fold increase, *p* < 0.0005), and PCL-HA30%/col (7.40-fold increase, *p* < 0.0001) scaffolds, than on PCL scaffolds (0.57 ± 0.09 absorbance at 405 nm/g scaffold (mean ± SD); Figure 8(d)).

## Discussion

PCL is a widely used 3D-printable biomaterial duo to its biodegradability, biocompatibility, slow degradation rate, and conducive mechanical characteristics.^31^ However, 3D-printed PCL scaffolds do not support cell attachment and/or proliferation due to its lack of bioactivity.^32^ PCL bioactivity can be promoted by coating PCL scaffolds with collagen and HA (PCL/col-HA)^23^ or by coating PCL-HA composite scaffolds with collagen (PCL-HA/col).^1^ Therefore, in this study we aimed to test which type of scaffold is more effective in stimulating osteogenic activity. Moreover, we characterized the scaffolds for their physicomechanical properties. We hypothesized that coating PCL scaffolds with collagen and HA or coating PCL-HA composite scaffolds with collagen have differential effects on the physicomechanical properties of 3D-printed PCL scaffolds, as well as the proliferation and osteogenic differentiation of pre-osteoblasts. We found that (i) The strand diameter was longer in PCL/col-HA and PCL-HA/col than in 3D-printed PCL scaffolds. (ii) 3D-printed PCL scaffolds exhibited a regular uniform structure with a smooth surface, while coating PCL scaffolds with collagen and HA or coating PCL-HA composite scaffolds with collagen changed the geometry, shape, and topography of the 3D-printed PCL scaffold surfaces; (iii) PCL/col-HA and PCL-HA/col scaffolds had higher hydrophilicity and surface roughness than 3D-printed PCL scaffolds; (iv) FE modeling revealed that the maximum von Mises stress at 2% compression strain remained below the yield stress of bulk material in all scaffold types, indicating that the scaffolds would not undergo irreversible deformation when in use; (v) PCL/col-HA scaffolds had a lower elastic modulus than 3D-printed PCL scaffolds, while PCL-HA/col scaffolds had a higher elastic modulus; (vi) PCL/col-HA scaffolds sharply enhanced ALP activity and calcium deposition compared to 3D-printed PCL scaffolds, while PCL-HA/col scaffolds most sharply enhanced cell proliferation and collagen production. Thus, our results indicated that cell-seeded PCL/col-HA scaffolds showed higher osteogenic differentiation potential than PCL-HA/col composite scaffolds in vitro, suggesting that 3D-printed PCL scaffolds with collagen-HA coating may be more promising for *in vivo* bone regeneration, especially in the short-term.

We found that 3D-printed PCL scaffolds had interconnected pores and a regular structure. Pore interconnection in 3D-scaffolds plays an important role in bone ingrowth, since it promotes oxygen transfer and cell distribution inside the scaffolds.^33^ Our findings indicated that PCL/col-HA and PCL-HA/col scaffolds had a larger strand diameter than 3D-printed PCL scaffolds. Additionally, we found that the average void size of the PCL/col-HA and PCL-HA/col scaffolds were in the range of 120-165 µm. Such a void size is optimal for cell proliferation and differentiation.^34^ As expected, the strand diameter in both PCL/col-HA and PCL-HA/col scaffolds was enhanced by increasing the HA concentration. Moreover, we found that PCL/col-HA and PCL-HA/col scaffolds had an irregular surface with little peaks and troughs, indicating that both collagen-HA coating and HA composite with collagen coating affects cell behavior on 3D-printed PCL scaffolds, as has been shown for surface-modified PCL scaffolds.^35^

Scaffold hydrophilicity is a critical factor for initial cell attachment and proliferation, as well as early bone apposition.^36^ Our results confirmed the hydrophobic nature of the 3D-printed PCL scaffolds, which was anticipated due to the hydrophobic tail present in PCL’s backbone.^37^ We showed that both PCL/col-HA and PCL-HA/col scaffolds had higher hydrophilicity than 3D-printed PCL scaffolds, which was also expected since both collagen and HA particles are highly hydrophilic, and therefore enhance the surface hydrophilicity of the scaffolds.^38^ Moreover, we showed that the hydrophilicity of the scaffolds increased by increasing the HA concentration. This was expected, as the addition of collagen and HA particles to PCL scaffolds introduces polar molecules to the surface, enhancing the hydrophilicity of the scaffold.^39^

PCL scaffolds typically degrade slowly via hydrolysis over a 6-24 months period.^40^ HA incorporation slightly accelerates this degradation due to increased hydrophilicity.^41^ Moreover, collagen coatings on PCL-HA/coll degrade (within weeks) through enzymatic action.^42^ For PCL/col-HA, the surface HA may enhance early degradation thereby promoting bone ingrowth, whereas PCL-HA/col could offer more controlled release of embedded HA during bulk degradation.

Surface roughness, on a micron scale, affects osteoblast attachment, viability, proliferation, and differentiation.^43^ We showed that PCL/col-HA and PCL-HA/col scaffolds had higher surface roughness than 3D-printed PCL scaffolds. Thus, 3D-printed PCL/col-HA and PCL-HA/col scaffolds have the potential to enhance cell attachment, viability, and osteogenic differentiation in vitro, similar to findings observed with surface-modified titanium featuring increased surface roughness.^44^ Our data showed that PCL/col-HA scaffolds had higher surface roughness than PCL-HA/col scaffolds, which might be explained by more HA particles on the surface of PCL/col-HA scaffolds than on PCL-HA/col scaffolds.

To support mechanical function, 3D-scaffolds must retain their structure post-implantation.^17^ The required high mechanical strength of scaffolds can be achieved by integrating reinforcing particles such as HA into polymer matrices.^45^ Our data showed that the compressive strength and elastic modulus of 3D-printed PCL-HA/col scaffolds were significantly higher than those of unmodified PCL scaffolds. This was expected since addition of HA particles in PCL scaffolds improves the mechanical properties of the PCL matrix.^46^ The elastic modulus of the scaffolds as calculated using FE-modeling was slightly higher than that obtained experimentally, which can be explained by the fact that FE-modeling did not consider the structural irregularity of the 3D-printed scaffolds. FE-modeling showed that increasing the HA concentration in both PCL/collagen-HA and PCL-HA/collagen scaffolds resulted in a more heterogenous distribution of von Mises stress. In addition, our results indicated that the maximum von Mises stress at 2% compression strain remained within the elastic region for all types of scaffolds, indicating it did not surpass the yield stress of bulk material. Hence, all scaffolds revealed suitable mechanical properties, *i.e*., elastic modulus, compressive strength, and von Mises stress, for bone tissue engineering applications.

Cell attachment and spreading are crucial for in vitro bone regeneration.^47^ We observed that pre-osteoblasts had a slightly spherical morphology on 3D-printed PCL scaffolds without surface functionalization, likely due to PCL hydrophobic nature and lack of biological recognition sites on its surface.^48^ Pre-osteoblasts exhibited a well-spread morphology on both PCL/col-HA and PCL-HA/col scaffolds, which can be attributed to increased hydrophilicity and surface roughness. Furthermore, cell seeding efficiency on all scaffolds was similar, indicating that a similar number of cells attached to the scaffolds during the first 8 h after cell seeding, probably as a result of the same 3D-architecture of all scaffolds, enabling cell entrapment within the scaffolds.^32^ Moreover, we found that cell proliferation was significantly increased on both PCL/col-HA and PCL-HA/col scaffolds compared to 3D-printed PCL scaffolds, but that the cell proliferation rate on the two scaffold types was similar. We also showed that by increasing the HA concentration, the cell proliferation on the scaffolds increased, which was expected since the HA particles play a key role in bone cell proliferation.^49^

A crucial factor for bone tissue engineering scaffolds is their capacity to promote bone regeneration.^50^ ALP activity, collagen matrix deposition, and calcium deposition on 3D-scaffolds are osteogenic markers illustrating pre-osteoblast activity.^51^ Our data showed higher expression of ALP, collagen matrix deposition, and calcium deposition in PCL/col-HA and PCL-HA/col scaffolds than in PCL scaffolds after 14 days of culture. This can be explained by increased surface hydrophilicity and roughness of the scaffolds, as well as by the presence of both collagen and HA particles, which are known to enhance osteogenic activity of pre-osteoblasts.^1^ Overall, both PCL/col-HA and PCL-HA/col scaffolds demonstrated improved bioactivity compared to unmodified PCL scaffolds. While PCL/col-HA most effectively enhanced ALP activity and calcium deposition indicative of osteogenic differentiation, PCL-HA/col promoted pre-osteoblast proliferation and collagen production. Since the ALP enzyme plays a role in bone mineral formation,^52^ these findings might indicate that PCL/col-HA scaffolds are more effective at promoting osteogenic differentiation compared to PCL-HA/col scaffolds.

The enhanced ALP activity and calcium deposition on PCL/col-HA scaffolds compared to PCL-HA/col scaffolds may stem from more uniform HA exposure and interaction with the cells on the scaffold surface. The HA directly interacts with pre-osteoblasts to promote differentiation via integrin-mediated signaling and activation of osteogenic pathways, *e.g*., Runx2.^53^ In contrast, PCL-HA/col scaffolds, with HA embedded in the PCL matrix, may provide a more stable collagen coating compared to PCL/col-HA scaffolds, that favors initial cell adhesion and proliferation through improved RGD-binding sites.^54^ This might result not only in increased collagen production, but also in a potential delay in HA exposure until minor scaffold degradation occurs.

We acknowledge that the focus of present study is on early osteogenic responses during a relatively short 14-days culture period, which aligns with our aim to assess possible short-term changes in pre-osteoblast activity. For longer-term differences, we hypothesize that PCL/col-HA scaffolds may continue to show superior mineralization due to better HA exposure promoting sustained calcium deposition, while PCL-HA/col scaffolds could support prolonged proliferation but potentially face issues with HA release during degradation, affecting matrix maturity. However, to address longer-term differences would require a separate study, *e.g*., up to 21-28 days.

## Conclusions

In this study, 3D-printed PCL scaffolds with collagen-HA coating containing 10, 20, or 30% (wt/wt) HA, as well as 3D-printed PCL-HA composite scaffolds containing 10, 20, or 30% (wt/wt) HA with collagen coating were successfully fabricated. PCL/col-HA and PCL-HA/col scaffolds had longer strand diameter, higher hydrophilicity, and increased surface roughness than PCL scaffolds. Both PCL/col-HA and PCL-HA/col scaffolds enahanced pre-osteoblast adhesion, proliferation, and osteogenic differentiation compared to PCL scaffolds. PCL-HA/col scaffolds were superior to PCL/col-HA scaffolds in promoting pre-osteoblast proliferation, but PCL/col-HA scaffolds had an important advantage over PCL-HA/col scaffolds in stimulating osteogenic differentiation of pre-osteoblasts, suggesting that PCL/col-HA scaffolds are more promising than PCL-HA/col scaffolds, especially in the short-term, to enhance osteogenic activity by pre-osteoblasts for *in vivo* bone regeneration.

## Data Availability

The data that support the findings of this study are openly available in figshare at https://figshare.com/s/8581fb3c2bde9578f274.
